# Inhibition of Inflammatory Mediators by Neobavaisoflavone in Activated RAW264.7 Macrophages

**DOI:** 10.3390/molecules16053701

**Published:** 2011-05-03

**Authors:** Ewelina Szliszka, Dariusz Skaba, Zenon P. Czuba, Wojciech Krol

**Affiliations:** 1 Department of Microbiology and Immunology, Medical University of Silesia in Katowice, Jordana 19, 41-808 Zabrze, Poland; Email: eszliszka@sum.edu.pl (E.S.); zczuba@sum.edu.pl (Z.P.C.); 2 Department of Conservative Dentistry and Endodontics Division of Dental Propedeutics, Medical University of Silesia in Katowice, Plac Akademicki 17, 41-902 Bytom, Poland; Email: darek@skaba.pl (D.S.)

**Keywords:** neobavaisoflavone, anti-inflammatory activity, RAW264.7 macrophages

## Abstract

Flavonoids and coumarins are the major bioactive constituents identified in *Psoralea corylifolia.* The active fraction isolated from fruits, seeds and roots possesses antibacterial, antioxidative and immunomodulatory properties. Neobavaisoflavone is one of the flavonoids found in *Psoralea corylifolia*. In the present study we investigated *in vitro* the anti-inflammatory activity of neobavaisoflavone. Macrophages play an important role in inflammation through the release of inflammatory mediators involved in the immune response. Inappropriate and prolonged macrophage activation is largely responsible for the pathology of acute and chronic inflammatory conditions. Neobavaisoflavone significantly inhibited the production of reactive oxygen species (ROS), reactive nitrogen species (RNS) and cytokines: IL-1β, IL-6, IL-12p40, IL-12p70, TNF-α in LPS+IFN-γ– or PMA– stimulated RAW264.7 macrophages.

## 1. Introduction

**Figure 1:**
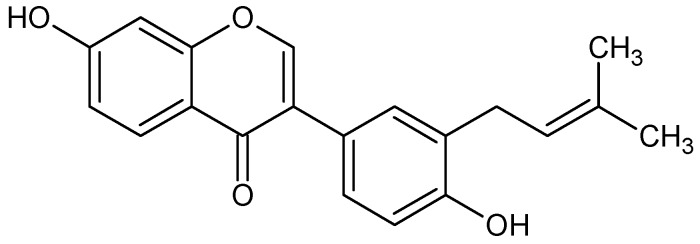
Chemical structure of neobavaisoflavone.

Neobavaisoflavone (7-hydroxy-3[4-hydroxy-3(3-methylbut-2-enyl)phenyl]chromen-4-one, Figure 1) belongs to the isoflavones, a subclass of the flavonoids. Flavonoids, as natural antioxidants, efficiently modulate redox status and inflammatory mediators production [1,2,3]. Our previous studies demonstrated the anticancer activity of neobavaisoflavone against LNCaP prostate cancer cells and HeLa cervical cancer cells [4,5,6]. Now we have tested for the first time the inhibition of inflammatory mediators in LPS+IFN-γ– or PMA–stimulated RAW264.7 murine macrophages by neobavaisoflavone.

Neobavaisoflavone was first isolated from the seeds of *Psoralea corylifolia* and later in a few *Erythrina* species [7,8,9,10,11,12]. The Leguminosae plant, *Psoralea corylifolia* L., is a medicinal plant widely distributed in India, China and Southeastern Asian countries. *Psoralea corylifolia* has traditionally been used for the treatment of leucoderma and other skin diseases, pollakiuria, nephritis, asthma, osteoporosis, hypertension and cardiovascular diseases [9,13,14]. The mature or dry fruits of this plant are a well known health supplement ingredient [15]. *In vitro* and *in vivo* studies have demonstrated that the active fraction from the fruits, seeds and roots of *Psoralea corylifolia* exhibits anticancer, antibacterial, antioxidative and anti-inflammatory properties [9,13,16,17,18]. The main active constituents identified from this herb are flavonoids (neobavaisoflavone, isobavachalcone, bavachalcone, bavachinin, bavachin, corylin, corylifol, corylifolin and 6-prenylnaringenin), coumarins (psoralidin, psoralen, isopsoralen and angelicin) meroterpenes (bakuchiol and 3-hydroxybakuchiol) and benzofuran glycosides (psoralenoside, isopsoralenoside) [7,8,12,14,15,19,20]. The variation of the neobavaisoflavone content in the tested samples was 1.59–2.96 mg/g [21]. 

Macrophages play an important role in inflammatory disease through the release of inflammatory factors such as reactive oxygen species (ROS), reactive nitrogen species (RNS), and cytokines involved in the immune response. Production of these macrophage mediators has been determined in many inflammatory tissues, following exposure to immune stimulants including bacterial endotoxin lipopolysaccharide (LPS) and interferon-gamma (IFN-γ). Inappropriate and prolonged macrophage activation is largely responsible for the pathology of acute (e.g. septic shock) or chronic inflammatory (e.g. rheumatoid arthritis, atherosclerosis, chronic hepatitis, pulmonary fibrosis) conditions [22,23]. 

Inhibition of inflammatory mediators’ production might be a useful therapeutic strategy in inflammatory diseases. There is an increasing interest in compounds isolated from herbal medications especially in chronic inflammatory diseases. Additionally to their pleiotropic immunomodulatory properties, they scavenge free radicals, are non-toxic, and pharmacologically safe [22,23,24]. 

This study was designed to investigate the *in vitro* effect of neobavaisoflavone on nitric oxide and pro-inflammatory cytokines production, and chemiluminescence in RAW264.7 activated macrophages.

## 2. Results and Discussion

Macrophages actively participate in inflammatory response by secretion of inflammatory mediators through pathogen– and host–derived molecules such as LPS and IFN-γ [1,23]. The RAW264.7 murine macrophage cell line is widely used to establish inflammatory models *in vitro* [1,2,22,23]. *Psoralea corylifolia* extract contains a number of bioactive compounds including flavonoids, coumarins, meroterpenes and benzofuran glycosides that are the molecular basis of its action [14,19,20]. Neobavaisoflavone is isolated from fruits and seeds of *Psoralea corylifolia* [7,8,14,15,19,20,21]*.* The biological activity of this isoflavone remains largely unknown.

RAW264.7 macrophage viability in the presence of neobavaisoflavone (1–100 μM) was measured by the MTT assay. Whereas the cytotoxicity of neobavaisoflavone against RAW264.7 cells was determined in LDH test. The results showed that neobavaisoflavone at the concentrations 1–100 μM had no effect on the viability and was not toxic to macrophages. The percentage of dead cells examined by MTT and LDH assays was near 0%. Therefore, for further studies of anti-inflammatory properties, we used neobavaisoflavone at the concentrations up to 100 μM. 

The inflammatory process is a complex of cellular and biochemical events leading to elimination of infectious agents. An ineffective or uncontrolled inflammatory response contributes to the cellular dysfunction, tissues damage that occurs in many chronic inflammatory diseases [23]. We investigated *in vitro* the effects of neobavaisoflavone on the generation of ROS, RNS and several cytokines in activated RAW264.7 macrophages. Nitric oxide (NO) mediates a variety of physiological and pathological processes including inflammatory reaction [25]. Macrophages are the major sources of inducible nitric oxide synthase (iNOS)– induced NO [26]. The increased production of NO is harmful to the host, leading to chronic inflammation condition or autoimmune diseases [22,23,25]. Therefore NO inhibition in inflammation has a potential therapeutic implication. 

**Figure 2:**
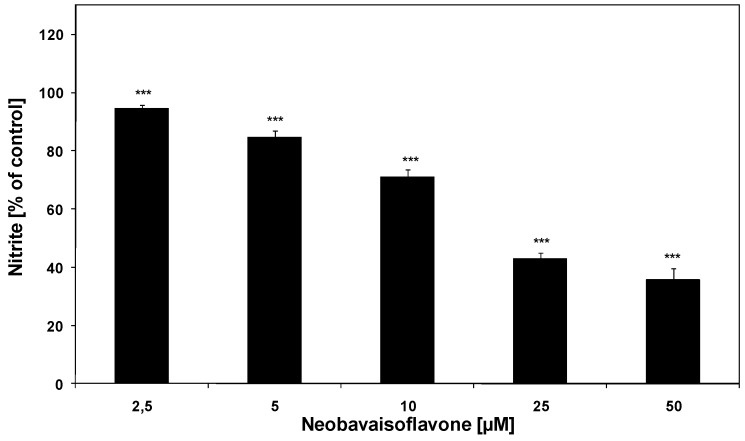
Effect of neobavaisoflavone on NO production in LPS plus IFN-γ– stimulated RAW264.7 macrophages. RAW264.7 cells were incubated with neobavaisoflavone at the concentrations of 1–50 μM for 20 hours. NO production was measured by the Griess reaction assay and expressed as a percentage of the control (LPS plus IFN-γ– stimulated cells). The values represent mean ±SD of three independent experiments (n = 12). *** = *P* < 0.001 compared to control (LPS plus IFN-γ).

**Figure 3:**
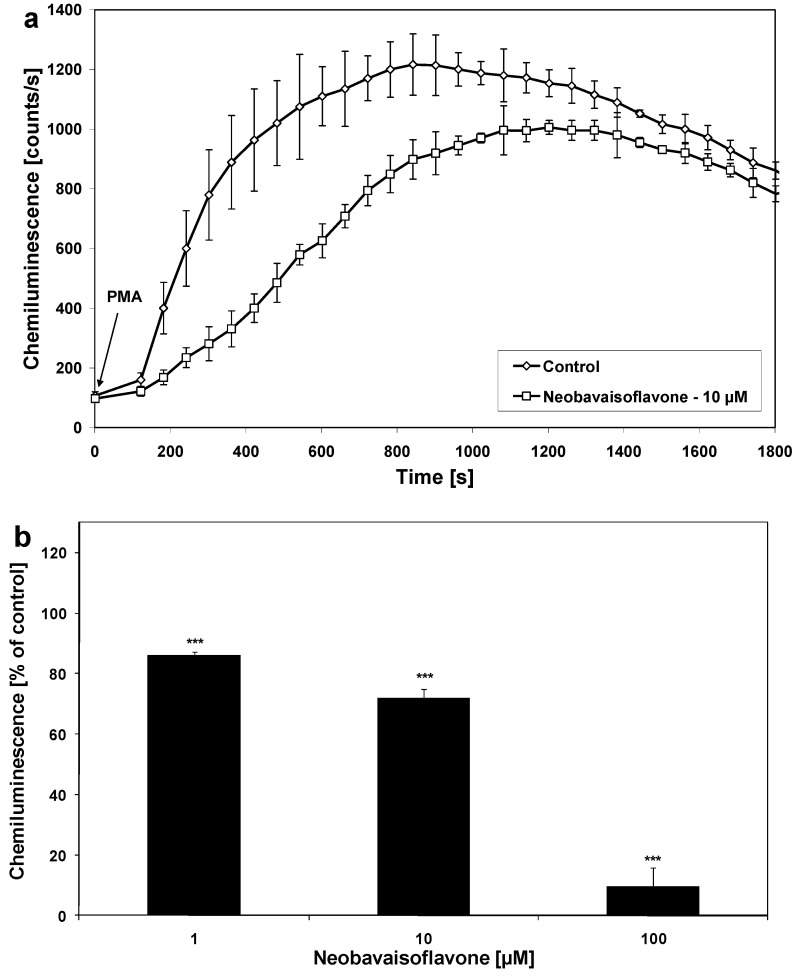
Effect of neobavaisoflavone on chemiluminescence of activated RAW264.7 macrophages. **(a) **Time course of chemiluminescence. **(b) **Chemiluminescence of PMA– stimulated RAW264.7 macrophages. The cells were incubated with neobavaisoflavone at the concentrations at 1–100 μM for 30 minutes. Chemiluminescence was determined using microplate luminometer and expressed as a percentage of the control (PMA– stimulated cells without neobavaisoflavone). The values represent mean ±SD of four independent experiments performed (n = 8). *** = *P* < 0.001 compared to control.

We found that neobavaisoflavone effectively decreased NO production in LPS plus IFN-γ– stimulated RAW264.7 macrophages. The effect of neobavaisoflavone on NO production in activated RAW264.7 cells is shown in Figure 2. The ED_50_ of neobavaisoflavone 25.0 μM (Table 1). Matsuda *et al.* demonstrated the inhibition of NO in LPS– stimulated mouse peritoneal macrophages by neobavaisoflavone (ED_50_ = 29.0 μM) [17]. Other isoflavones, such as genistein and daidzein also inhibited NO production in activated RAW264.7 cells [22]. Changes in production of ROS and RNS production were determined by a chemiluminescence assay [27,28]. Neobavaisoflavone decrease the chemiluminescence in PMA– stimulated RAW264.7 macrophages (Figure 3) with an ED_50_ of 19.94 μM as measured by a microplate luminometer in activated RAW264.7 cells. 

**Figure 4:**
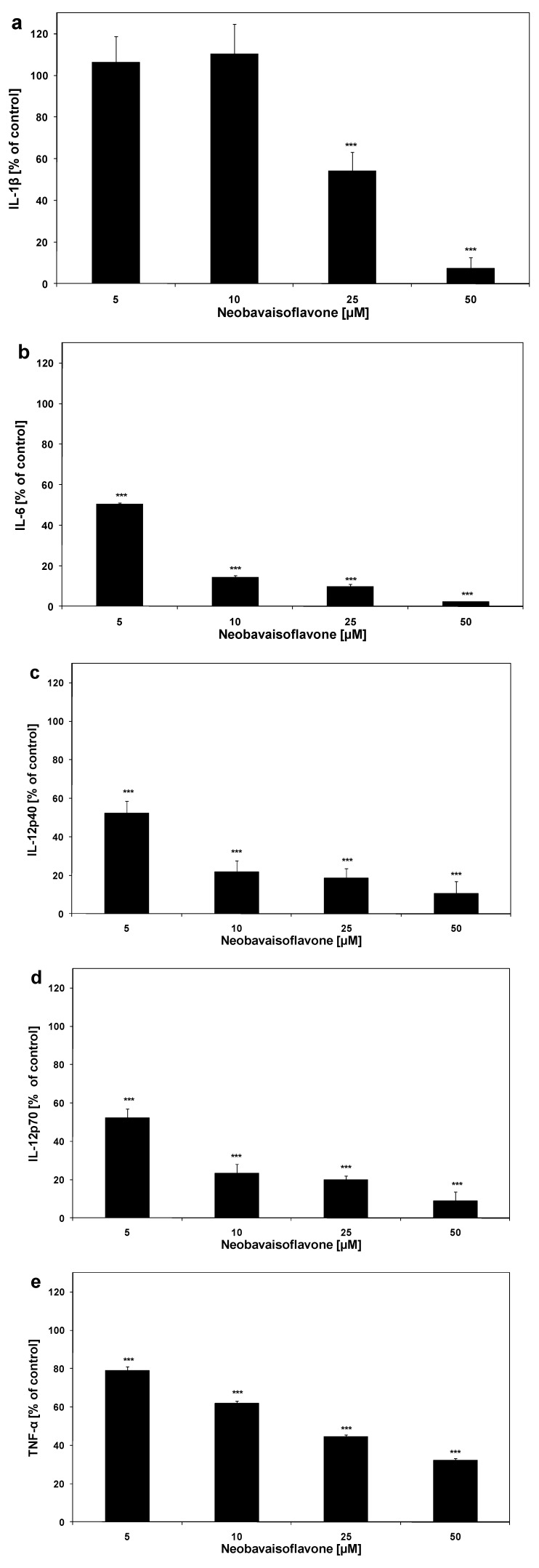
Effect of neobavaisoflavone on cytokines production in LPS plus IFN-γ– stimulated RAW264.7 macrophages: **(a)** IL-1β, **(b)** IL-6, **(c) **IL-12p40, **(d) **IL-12p70, **(e) **TNF-α. RAW264.7 cells were incubated with neobavaisoflavone at the concentrations of 1–50 μM for 20 hours. Cytokine concentrations in the culture medium were determined by Multiplex bead-based cytokine assay. The values represent mean ±SD of two independent experiments performed (n = 8). *** = *P* < 0.001 compared to control (LPS plus IFN-γ).

The cytokines’ release was measured simultaneously by a Bio-Plex suspension array system. This assay is designed for the multiplexed quantitative measurement of multiple cytokines in a single well using 50 μL of sample. IL-1β, IL-6, IL-12 and TNF-α are critical in the inflammatory network. The effect of neobavaisoflavone on cytokine production in LPS plus IFN-γ activated RAW264.7 cells is shown in Figure 4

The multifunctional cytokine IL-6 takes part in hematopoiesis, regulation of immune response, and inflammation. Increased IL-6 levels are often in rheumatoid arthritis, Crohn’s disease, psoriasis, and encephalomyelitis [29]. Thus, inhibitors of IL-6 synthesis are useful in the treatment of autoimmune diseases. Neobavaisoflavone (ED_50_ = 5.03 μM) significantly inhibited IL-6 release in activated RAW264.7 macrophages. The cytokine IL-12, a heterodimer of the p40 and p70 subunits is secreted by macrophages, dendritic cells and lymphocytes B. IL-12p40 induces production of NO and TNF-α [30]. Neobavaisoflavone significant inhibited IL-12p40 and IL-12p70 expression in stimulated RAW264.7 macrophages (ED_50_ = 5.23 μM and ED_50_ = 5.26 μM, respectively). TNF-α was reported to induce the release of IL-1, IL-6, and NO. NO could promote TNF-α production in mouse macrophage in turn. The data demonstrated the significance of TNF-α in the pathological damage of inflammation and septic shock [31]. Neobavaisoflavone downregulated the production of TNF-α (ED_50_ = 18.80 μM). IL-1β is the key inflammatory cytokine produced after stimulation of macrophage and many other cell type by almost all infectious factors. The release of IL-1β by activated RAW264.7 is decreased by neobavaisoflavone (ED_50_ = 23.11 μM). 

Kole *et al.* have reported that the isoflavone biochanin-A showed anti-inflammatory activities through inhibition of IL-1β, IL-6, TNF-α, and NO production with decreased expression of iNOS in LPS–stimulated RAW264.7 [26]. The ED_50_ values of neobavaisoflavone on cytokine production in activated macrophages RAW264.7 are shown in Table 1. The flavonoid at concentrations of 5 – 25 μM significantly inhibited the production of IL-1β, IL-6, IL-12p40, IL-12p70, TNF-α in LPS + IFN-γ –stimulated RAW264.7 cells.

**Table 1:** Effect of neobavaisoflavone on NO and cytokine production in LPS+IFN-γ –stimulated RAW264.7 macrophages.

Inflammatory mediator	ED_50_ [μM]
NO (nitrite)	25.0
IL-1β	23.11
IL-6	5.03
IL-12p40	5.23
IL-12p70	5.26
TNF-α	18.80

## 3. Experimental

### 3.1. General

The neobavaisoflavone was obtained from Alexis Biochemicals (San Diego, CA, USA). The compound was dissolved in dimethyl sulphoxide (DMSO) to obtain the working concentration solutions. Lipopolysaccharide (LPS *E.coli* O111:B4) was purchased from Fluka Chemie GmbH (Buchs, Switzerland) and recombinant mouse interferon-gamma (IFN-γ) was purchased from R&D Systems (Minneapolis, MN, USA). 

### 3.2. Cell Culture

Murine peritoneal macrophage cell line RAW264.7 was obtained from the ATCC (American Type Culture Collection, Manassas, VA, USA). The cells were cultured in RPMI 1640 medium supplemented with 10% heat inactivated fetal bovine serum, 4 mM L-glutamine, 100 U/mL penicillin, and 100 μg/mL streptomycin and maintained in monolayer cultures at the temperature 37 °C and atmosphere containing 5% CO_2_ [1]. Reagents for cell culture were purchased from ATCC. RAW264.7 were seeded at a density of 1 × 10^6^/mL cells (2 × 10^5^/well) in 96-well plates at the presence of LPS (200 ng/mL) and IFN-γ (25 U/mL) with or without neobavaisoflavone for 20 hours.

### 3.3. Cell Viability Assay

The cell viability was determined by 3-(4,5-dimethyl-2-thiazyl)-2,5-diphenyl-2*H-*tetrazolium bromide (MTT) reduction assay as described in the literature [32,33]. The RAW264.7 cells (1 × 10^6^/mL) were seeded 3 hours before the experiments in a 96-well plate. Various combinations of neobavaisoflavone (1, 2.5, 5, 10, 25, 50, 100 μM) with or without LPS+IFN-γ were added to the cells. Final volume was 200 µL. After 20 hours the medium was removed, and MTT solutions (20 μL, 5 mg/mL, Sigma Chemical Company, St. Louis, MO, USA) were added to each well for 4 hours. The resulting formazan crystals were dissolved in DMSO. Controls included native cells and medium alone. The spectrophotometric absorbance at 550 nm was measured using an ELx 800 microplate reader (Bio-Tek Instruments Inc., Winooski, VT, USA). The viability was calculated by the formula: percent of viable cells = (absorbance of experimental wells/absorbance of control wells) × 100%. 

### 3.4. Lactate Dehydrogenase Release Assay

Lactate dehydrogenase (LDH) is a stable cytosolic enzyme released upon membrane damage in necrotic cells. LDH activity was measured using a commercial cytotoxicity assay kit (Roche Diagnostics GmbH, Mannheim, Germany). The RAW264.7 (1 × 10^6^/mL) cells were treated with various combinations of neobavaisoflavone (1, 2.5, 5, 10, 25, 50, 100 μM) with or without LPS+IFN-γ for the indicated period of time. LDH released in culture supernatants is detected with coupled enzymatic assay, resulting in the conversion of a tetrazolium salt into a red formazan product. The maximal release was obtained after treating control cells with 1% Triton X-100 (Sigma) for 10 minutes at room temperature. The spectrophotometric absorbance at 490 nm was measured using the ELx 800 microplate reader [34,35]. The necrotic percentage was expressed using the formula: (sample value/maximal release) × 100%.

### 3.5. Quantification of Nitric oxide Production

Macrophages RAW264.7 stimulated with LPS+IFN-γ were incubated with various concentrations of neobavaisoflavone (1, 2.5, 5, 10, 25, 50 μM) for 20 hours. After this time nitric oxide (NO) production was determined by measuring the accumulation of nitrite, a stable end product, in the culture supernatant according to the Griess reaction [25]. Equal volumes of culture supernatant from each well or medium (100 μL) was mixed with 100 μL of Griess reagent in a 96-well plate and incubated for 15 minutes at room temperature. The spectrophotometric absorbance was read at 550 nm in the ELx 800 microplate reader and the nitrite concentration in the medium was calculated using sodium nitrite as a standard. Nitrite was not detectable in cell-free medium.

### 3.6. Detection of ROS and RNS Production by Chemiluminescence

Chemiluminescence of the macrophages RAW264.7 was evaluated by microplate method in Hank’s balanced salt solution, pH 7.4, at room temperature. The cells were incubated with neobavaisoflavone at the concentrations of 1, 10, 100 μM for 30 minutes. Next luminol (Sigma) solution was added to wells containing 2 × 10^5^ cells, giving a final concentration of 1.13 × 10^−4^ M. After 5 minutes, for macrophages’ stimulation phorbol 12-myristate 13-acetate (PMA) solution (Sigma) was injected to obtain the concentration of 8 × 10^−7^ M. The final volume of each sample was 200 μL. The chemiluminescence was determined for 5 minutes with luminol alone and after stimulation with PMA for 30 minutes. The measuring system was equipped with LB 960 CentroXS^3^ microplate luminometer (Berthold Technologies GmbH, Wildbad, Germany) [27,28,36].

### 3.7. Multiplex Bead-Based Cytokine Assay

Cytokines released from treated RAW264.7 cells were determined in the cell culture supernatants with a Pro Mouse Cytokines 5-plex assay kit for IL-1β, IL-6, IL-12p40, IL-12p70, TNF-α (Bio-Rad Laboratories Inc., Hercules, CA, USA). This assay was performed using Bio-Plex 200 System based on xMAP suspension array technology (Bio-Rad). The LPS+IFN-γ stimulated and native (control) RAW264.7 cells (1 × 10^6^/mL) were incubated with or without neobavaisoflavone at the concentrations of 1, 2.5, 5, 10, 25, 50 μM for 20 hours. Standard curves for each cytokine were generated using kit-supplied reference cytokine sample. The assay used in these experiments is designed for the multiplexed quantitative measurement of multiple cytokines in a single well using 50 μL of sample. Briefly, the following procedure was performed: after pre-wetting the 96-well filter plate with washing buffer, the solution in each well was aspirated using a vacuum manifold. Next, cell culture supernatants were incubated with antibody-conjugated beads for 30 minutes. Following the incubational period, detection antibodies and streptavidin-PE were added to each well for 30 minutes. Then, after washing with buffer to remove the unbound streptavidin-PE, the beads bound to each cytokine were analysed in the Bio-Plex 200 instrument. The fluorescence intensity were evaluated using Bio-Plex Manager software (Bio-Rad) [23].

### 3.8. Statistical Analysis

The values represent mean ±SD of two, three or four independent experiments performed in duplicate or quadruplicate. Significant differences were analyzed using Student’s t-test and *P*-values < 0.05 were considered significant. The concentration-response curves were analysed using Pharma/PCS version 4 (Pharmacological Calculations System) software.

## 4. Conclusions

Neobavaisoflavone significantly inhibited the production of ROS, RNS, and cytokines: IL-1β, IL-6, IL-12p40, IL-12p70, TNF-α in activated RAW264.7 macrophages, demonstrating the anti-inflammatory activity of this compound.
